# Supplementary material for: C-State: an interactive web app for simultaneous multi-gene visualization and comparative epigenetic pattern search
Source: BMC Bioinformatics. 2017 Sep 13;18(Suppl 10):392. doi: 10.1186/s12859-017-1786-6 (PMC5606219; doi:10.1186/s12859-017-1786-6)
Supplement: Additional file 1: Figure S1. — Screenshot of the Filters Panel in the pattern search module showing all the 7 filters available (left) and the 4 filters opened in the Active Filters pane on the right. Figure S2. Table view listing genes having bivalent domains at promoters in ESCs (13 of 330 genes). Figure S3. Feature counts filters set to identify genes bivalent in ESCs that show a promoter (−5 Kb to +2 Kb of TSS) profile of A) H3K27me3 marks but no H3K4me3 enrichment in GM12878 cells and B) H3K4me3 peaks but no H3K27me3 enrichment in K562 cells. Figure S4. Top: Feature Overlaps filter set to identify genes in ESCs that carry H3K36me3 enrichment at exons (within 0.5 Kb) indicating active transcription. Bottom: Gene Expression filter added to the chain to identify genes that additionally have high transcript levels. Figure S5. Gene Expression scatterplot (Plots and Analysis) showing distribution of expression values of the 97 filtered genes obtained after setting the filter described in Fig. 4, top. Many of these genes appear to be ESC-specific as they show medium to high expression in ESCs (boxed, column 2, X-axis represents expression in ESCs) compared to the other cell types. Figure S6. View accordion displaying the genes filtered for high gene expression only in ESCs. Video demos are available from the C-State website. (DOCX 720 kb) [file 12859_2017_1786_MOESM1_ESM.docx]

**Additional file 1**
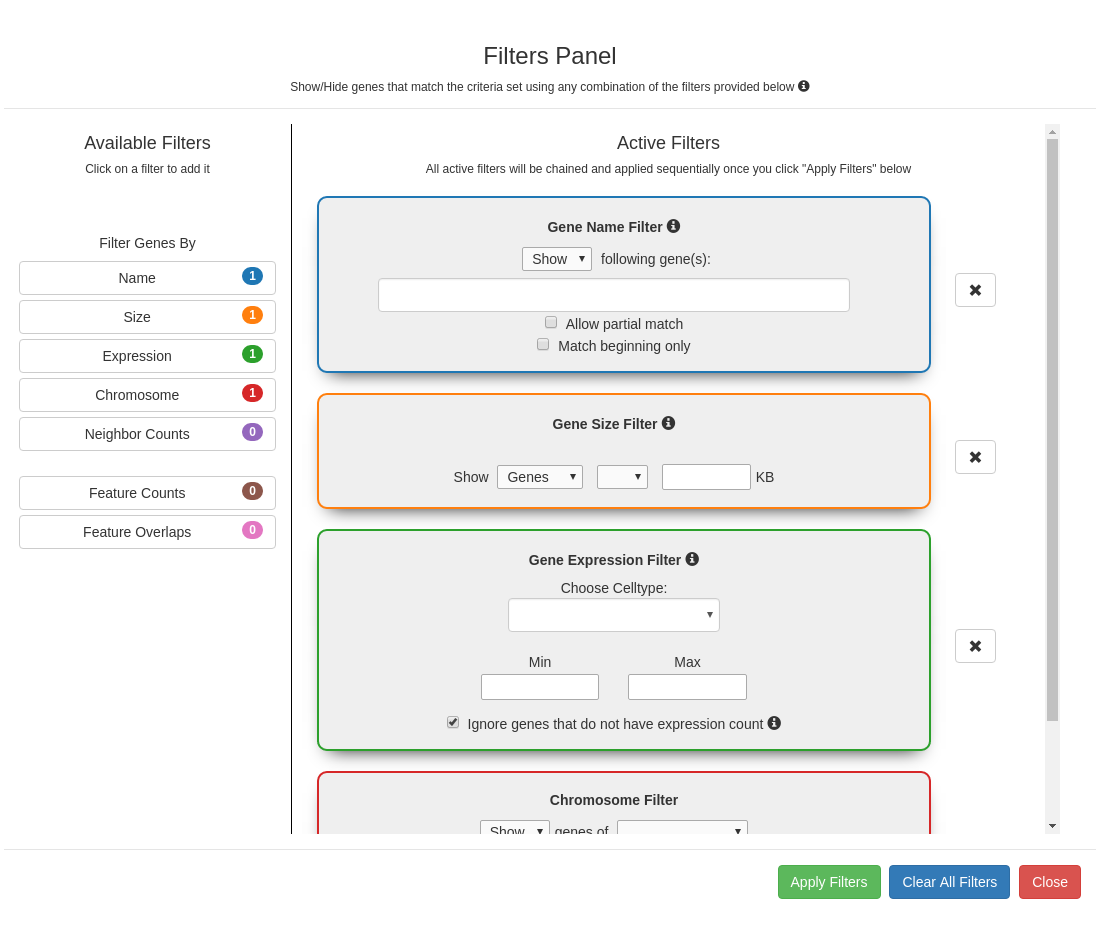


**Figure S1:** Screenshot of the Filters Panel in the pattern search module showing all the 7 filters available (left) and the 4 filters opened in the Active Filters pane on the right.


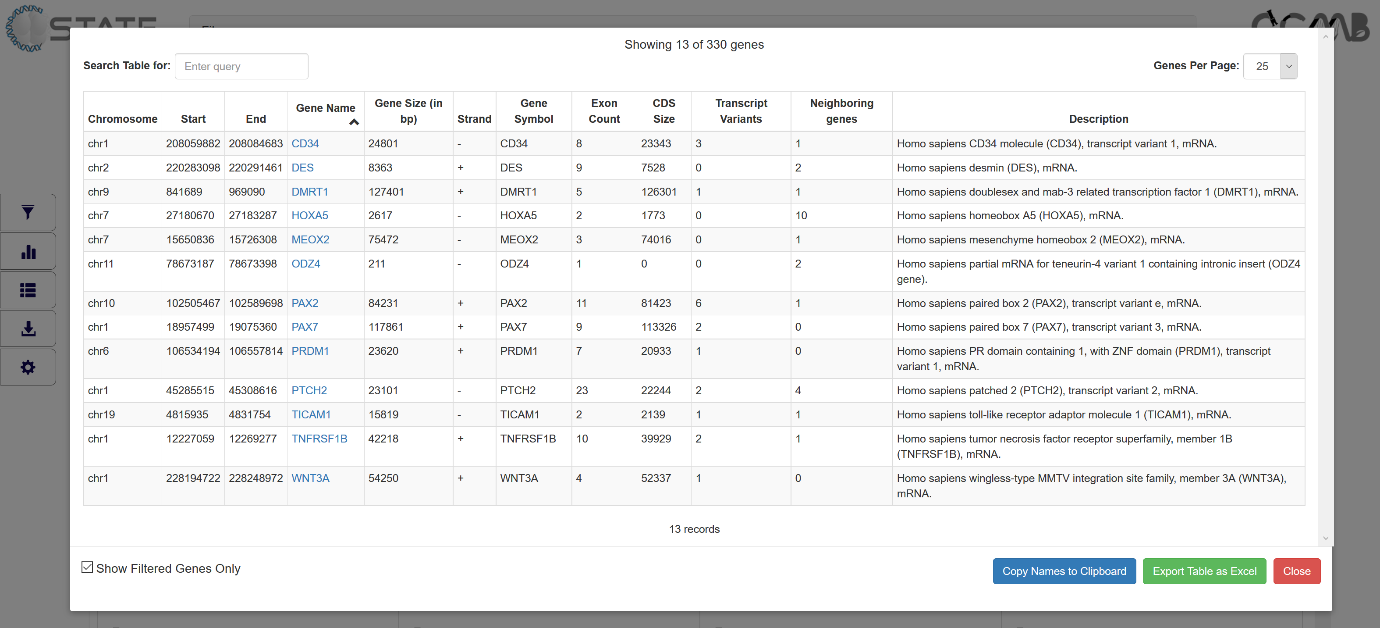


**Figure S2:** Table view listing genes having bivalent domains at promoters in ESCs (13 of 330 genes)

**3A**


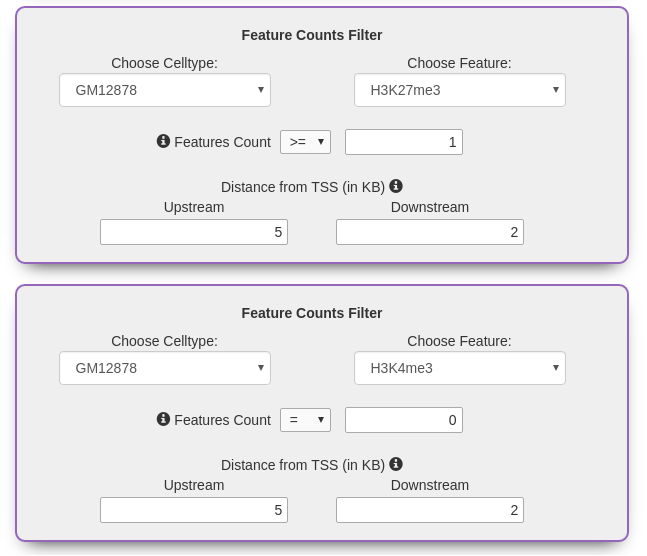


**3B**


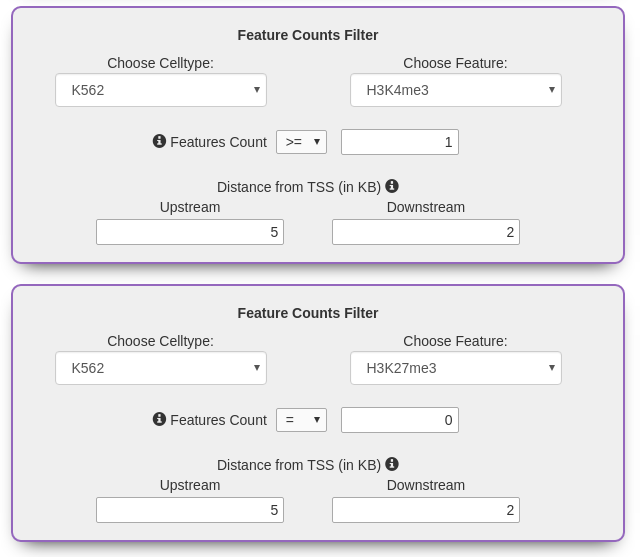


**figure S3:** Feature counts filters set to identify genes bivalent in ESCs that show a promoter (-5 Kb to +2 Kb of TSS) profile of **A)** H3K27me3 marks but no H3K4me3 enrichment in GM12878 cells and **B)** H3K4me3 peaks but no H3K27me3 enrichment in K562 cells


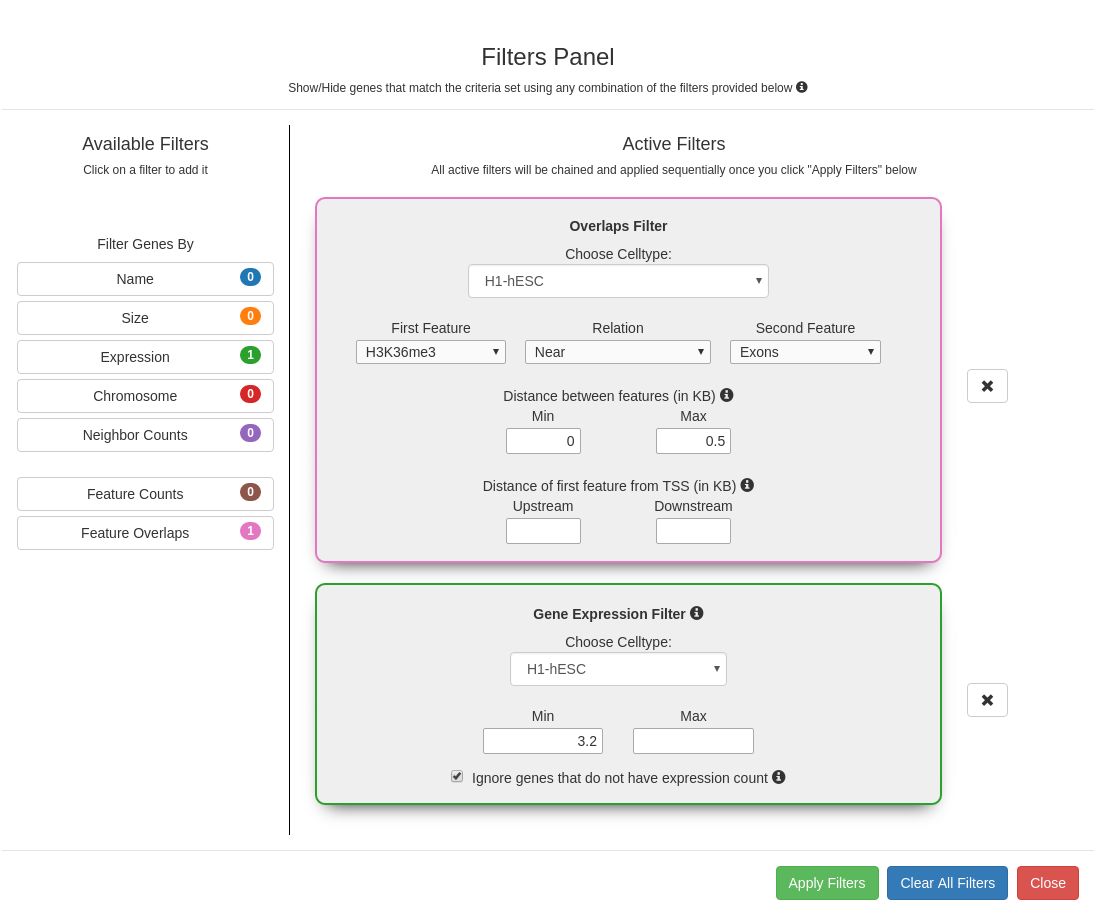

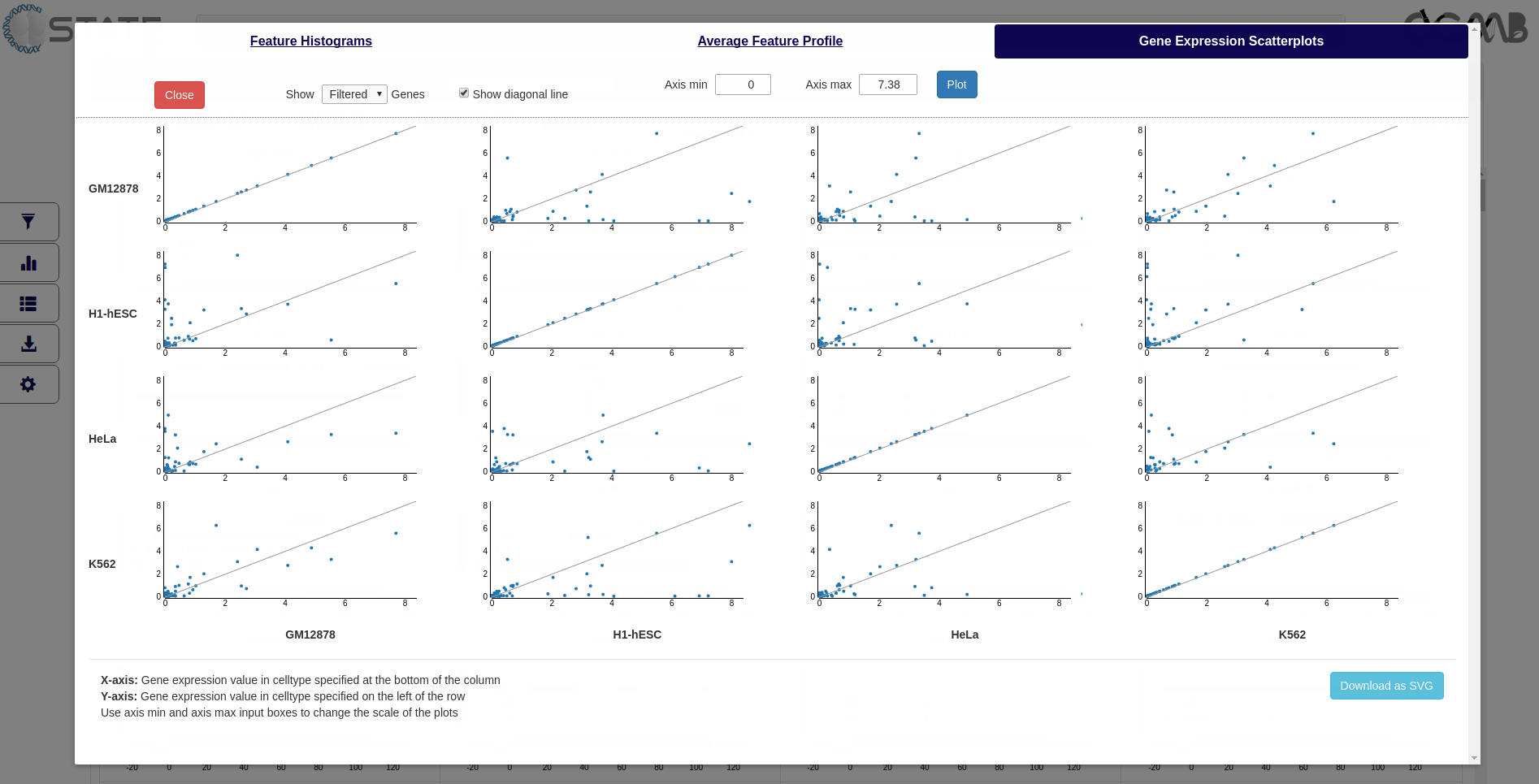


**figure S4:** Top: Feature Overlaps filter set to identify genes in ESCs that carry H3K36me3 enrichment at exons (within 0.5 Kb) indicating active transcription. Bottom: Gene Expression filter added to the chain to identify genes that additionally have high transcript levels.

**figure S5:** Gene Expression scatterplot (Plots and Analysis) showing distribution of expression values of the 97 filtered genes obtained after setting the filter described in Figure 4, top. Many of these genes appear to be ESC-specific as they show medium to high expression in ESCs (column 2, rows 1, 3 & 4, bottom right to the diagonal line) compared to the other cell types.


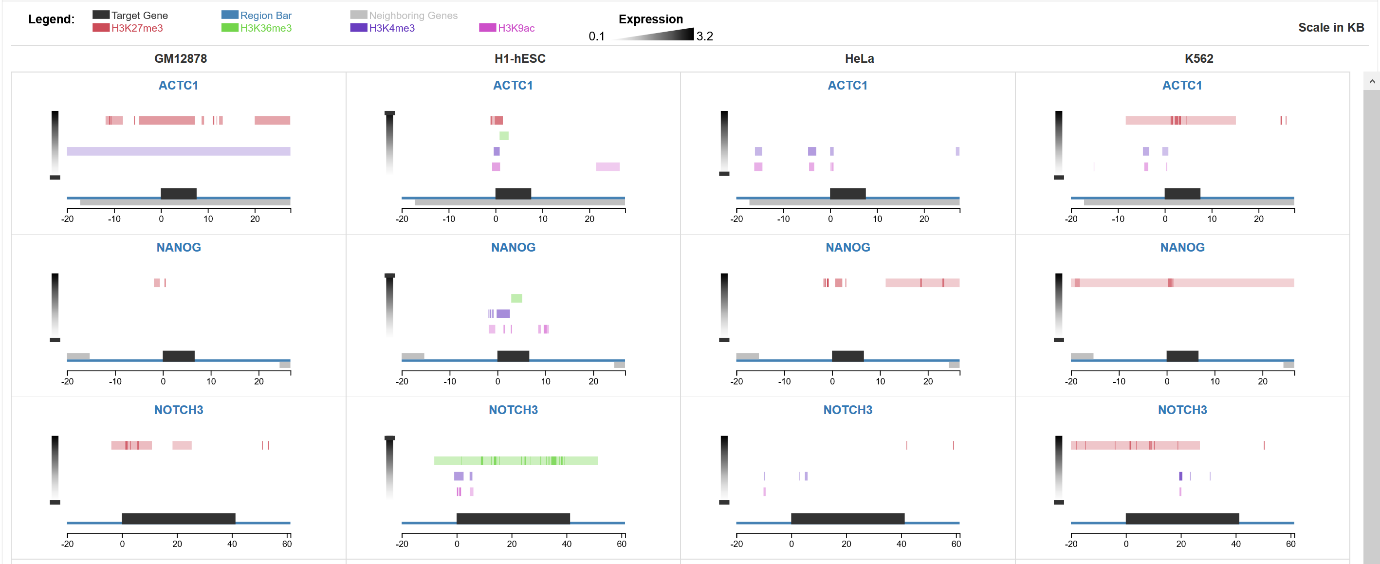


**figure S6:** View accordion displaying the genes filtered for high gene expression only in ESCs
